# HAMAgent: human assisted multiagent system for emotion recognition and digital health—a survey and preliminary study

**DOI:** 10.3389/fdgth.2026.1803433

**Published:** 2026-07-09

**Authors:** Yupei Li, Qiyang Sun, Jiahao Xue, Aydin Javadov, Manuel Milling, Weitong Zhang, Bernhard Kainz, Björn W. Schuller

**Affiliations:** 1 GLAM—Group on Language, Audio, & Music, Imperial College London, London, United Kingdom; 2 CHI—Chair of Health Informatics, TUM University Hospital, Munich, Germany; 3 School of Engineering and Materials Science, Queen Mary University of London, London, United Kingdom; 4 Eidgenössische Technische Hochschule Zürich, Zurich, Switzerland; 5 Human in the Loop (HITL), Imperial College London, London, United Kingdom

**Keywords:** digital health, emotion recognition, human in the loop, large language models, multiagent

## Abstract

This study surveys existing multi-large language model (LLM) agent applications, comparing systems that incorporate active human participation with those that operate fully autonomously within digital health contexts. Building on this analysis, we conduct an early exploration of human-in-the-loop feedback in multi-LLM interactions for emotion and human behaviour understanding using a subset of the FairytaleQA dataset. We further propose the HAMAgent framework to investigate how human feedback influences multi-agent reasoning and performance. Our preliminary experiments demonstrate the potential benefits of integrating human guidance into multi-LLM workflows and provide insights for designing effective human-in-the-loop systems in digital health. We conclude by discussing the key implications of our findings and outlining future directions for multi-agent LLM systems enhanced with human input.

## 1 Introduction

Large language models (LLMs) have recently demonstrated strong general-purpose capabilities across a wide range of domains. However, they still face significant challenges in tasks that require understanding or alignment with human values, such as those in affective computing [1, 2] and digital health [3]. These limitations are closely tied to broader concerns about whether artificial intelligence (AI) systems can be safely endowed with the ability to comprehend and act upon human values [4].

Despite these concerns, there is increasing interest in leveraging AI including LLMs within digital health, provided that their use is carefully controlled to avoid risks such as privacy leakage or inappropriate substitution for clinicians [5]. When deployed responsibly, LLMs offer several potential benefits: they can generate suggestions informed by large-scale training data, support more holistic assessments, and streamline clinical workflows, for example by assisting with medical reports.

However, two major challenges remain in the utilisation of LLMs for digital health. The first concern is their limited grounding in human values and domain knowledge. While LLMs should not be positioned as replacements for humans, it is essential to incorporate human expertise such as empathy, psychological understanding, and contextual judgment into these systems to support more accurate and trustworthy decision-making. Prior work has attempted to align models with such forms of knowledge [6, 7], yet, substantial scaling and refinement are still needed before these approaches can meet the requirements of practical digital health applications.

Another major concern is the difficulty of generalising across the diverse set of tasks required in real-world clinical and behavioural settings. Healthcare environments encompass a wide range of responsibilities such as therapist diagnosis, nurse supervision of patient conditions, and administrative decision-making, each demanding specialised expertise. Although LLMs exhibit strong general-purpose capabilities, there is an increasing need for domain-specialised models to ensure higher quality and reliability for individual tasks [8]. To manage systems involving multiple complex tasks, multi-agent LLM architectures have been proposed as a promising solution [9]. In such systems, different LLMs are assigned specific roles such as solvers for domain-specific subtasks or judges that evaluate and coordinate outputs and interact through structured communication to produce a final decision. However, even with advances in multi-agent frameworks [10], these systems still require the integration of human values to ensure that their outputs are trustworthy and persuasive to patients.

To address the two gaps outlined above, our work offers both a conceptual and early exploration of empirical contributions. First, we provide an overview of existing multi-LLM systems in digital health, contrasting frameworks that incorporate human participation with those that operate autonomously. Building on these insights, we propose HAMAgent, a human-in-the-loop (HITL) multi-agent framework designed to integrate human knowledge and values into LLM interactions while supporting iterative self-improvement through structured feedback. Together, these contributions aim to inform the development of more reliable, human-aligned multi-LLM systems for digital health applications.

## 2 Related works

**Figure 1:**
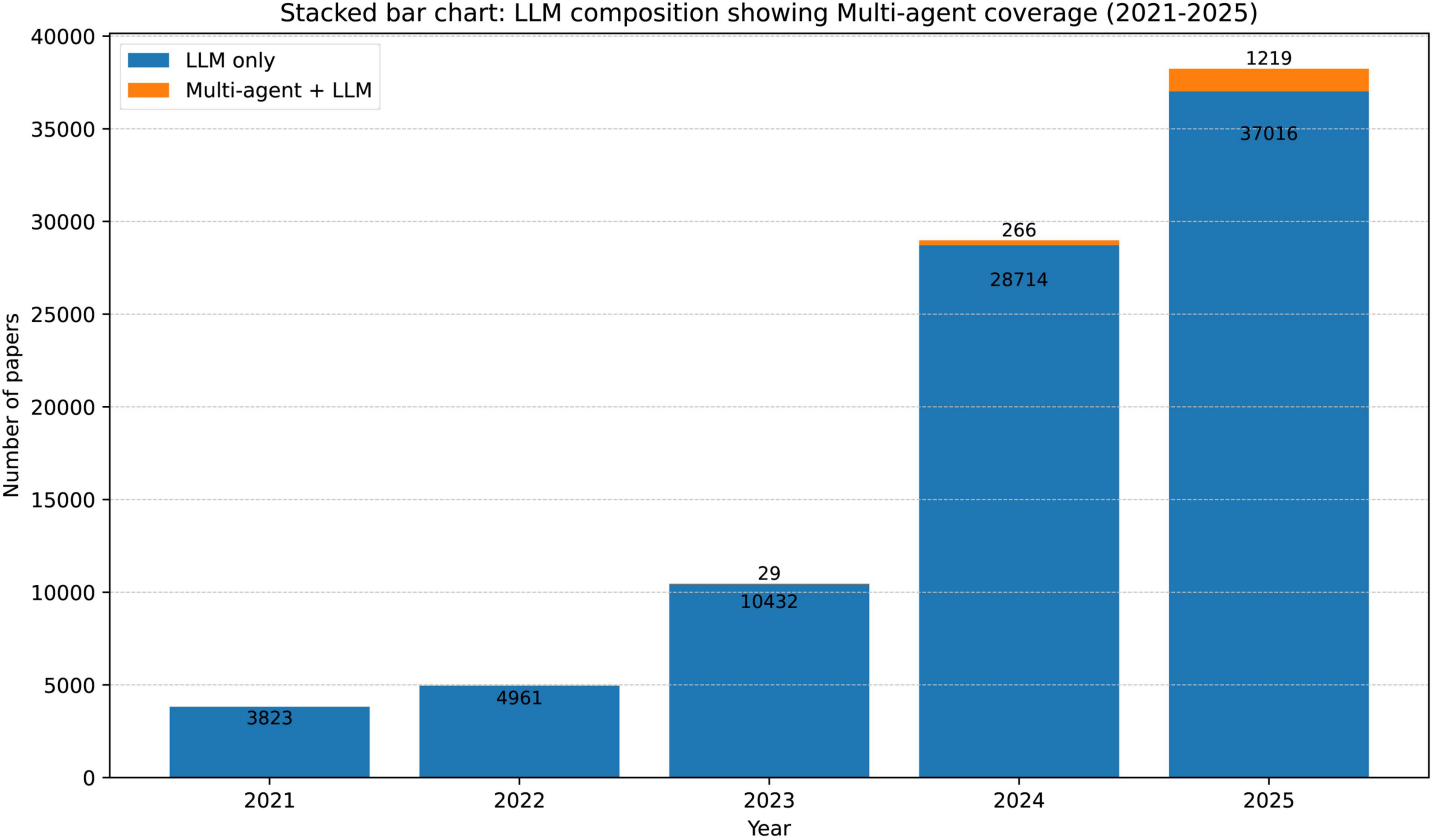
Paper numbers from the Scopus database between 2021–2025 with key words LLM or Multi-agent and LLM.

Multi-agent systems have attracted considerable research interest in recent years due to their strong capability to address a wide variety of complex problems. Over the past five years, the number of related publications has steadily increased, as illustrated in Figure 1. Notably, multi-agent systems that incorporate LLMs have emerged as a rapidly developing research direction. The figure also indicates the growing prominence of LLMs themselves within the research community. However, there are only 3 papers discussing LLMs with multiagent systems in digital health, showing the need for exploration on this topic. We conduct this thematic review as both a foundation for understanding the current landscape and an invitation for the research community to further explore and advance this emerging topic.

### 2.1 Single LLM systems

To begin with, we examine single-LLM systems for task solving in order to understand why they are often disadvantaged compared to multi-agent systems.

In principle, LLMs exhibit broad general-purpose capabilities and are widely regarded as promising steps toward artificial general intelligence [11]. However, this generality constitutes a double-edged sword. When a single LLM is trained to perform multiple heterogeneous tasks, the optimisation process becomes highly complex, particularly with respect to credit assignment. For instance, LLMs may be trained simultaneously for financial portfolio allocation, order execution, and related decision-making tasks [12]. When such a model fails to produce the expected output, it is difficult to identify which component of the underlying reasoning or decision process is responsible for the failure. In contrast, multi-agent systems explicitly decompose the overall task into specialised sub-tasks, assigning each to a dedicated agent [13]. This structural separation enables clearer credit assignment, more targeted optimisation, and more interpretable error analysis. In principle, this should lead to more consistent gradients without multitask gradient inference [14]. This leads to specialised LLMs employed as task-specific agents often outperforming general-purpose LLMs [15], providing further empirical evidence of the aforementioned optimisation and credit assignment challenges inherent in monolithic models.

Catastrophic forgetting poses another challenge for single-LLMs when they are required to cover multiple tasks [16]. Although many tasks share common capabilities such as general language understanding and reasoning, they also demand task-specific skills that can benefit from fine-tuning on different datasets [17]. Sequential optimisation on heterogeneous data distributions inevitably interferes with previously learnt representations, leading to the erosion of earlier knowledge. This phenomenon is a well-known limitation of continual and multi-task learning, and is particularly pronounced in LLMs, which rely on a single shared parameter space to support a wide range of tasks [18]. The theory of mixture-of-experts (MoE) models [19] proposes a principled solution to this challenge. Although MoE architectures do not constitute true multi-agent systems, they provide direct evidence that partitioning tasks across specialised experts can effectively mitigate forgetting. This insight aligns closely with the multi-agent paradigm, where task decomposition and specialisation across agents serve as a potential solution to the limitations of single-LLM systems.

At a level beyond optimisation, single LLMs often struggle with effective cognitive decomposition. When confronted with complex tasks that require multi-step solutions such as mathematical reasoning, the model must simultaneously perform question understanding, logical inference, calculation or proof construction, and final answer generation within a single forward process. This requirement forces all cognitive sub-processes to be entangled within one inference trajectory, making the reasoning path difficult to control or verify. The introduction of chain-of-thought [20] prompting provides clear evidence of this limitation, as it explicitly encourages the model to decompose a problem into intermediate steps. However, in principle, chain-of-thought merely expands a single cognitive stream linearly, without introducing explicit modularisation or dynamic interaction among distinct reasoning components. This challenge has also been described in the literature as context interference [21]. Different contextual inputs often emphasise distinct aspects of a task, leading to divergent model outputs. However, because LLMs operate as autoregressive generation models, they are inherently sensitive to such contextual variations, making them particularly vulnerable to interference among heterogeneous context elements. In contrast, multi-agent systems mitigate context interference by isolating contextual information across specialised agents and enabling structured communication among them. This separation reduces unintended interactions within a single context window while allowing relevant information to be selectively exchanged between agents.

### 2.2 LLM interactions

As discussed above, single-LLM systems exhibit several inherent limitations. Motivated by these drawbacks, we investigate interaction mechanisms in multi-agent LLM systems. In general, such systems comprise five typical roles played by LLMs. Beyond the *solver*, which is responsible for addressing the core problem and very alike specialised LLMs, the system may include a *planner*, *critic*, *refiner*, and *retriever*. Each role may be instantiated by one or multiple LLMs, depending on the task complexity and system design.

The *planner* decomposes a complex task into a set of subtasks and orchestrates their execution, effectively acting as a task allocator and system manager [22]. By performing high-level problem understanding and pre-screening, the planner provides a structured execution plan for downstream agents. Some related frameworks, however, such as CAMEL, do not include explicitly a planner role. We argue that human input in such systems can be viewed as fulfilling this function, highlighting that humans may also be considered integral components of multi-agent systems [23].

The *critic* evaluates the performance of the solver and provides feedback on the quality, correctness, or consistency of its outputs, a paradigm commonly referred to as *LLMs as judges* [24]. When combined with a *refiner* such as in the RoCo system [25], the critic enables iterative improvement by guiding answer revision based on identified errors or weaknesses. This process is conceptually related to self-consistency methods [26], but extends them by introducing explicit role separation and feedback loops. More broadly, the critic plays a crucial role in reducing error propagation and improving robustness, as evaluation and feedback mechanisms are fundamental components of effective multi-agent systems [27].

The *retriever* serves as an auxiliary, yet important component when addressing complex tasks. It is responsible for retrieving relevant external information to support reasoning and decision-making, thereby improving both efficiency and effectiveness. Retrieval mechanisms are commonly instantiated through retrieval-augmented generation (RAG) frameworks, which enable LLMs to ground their outputs in task-relevant knowledge and reduce reliance on parametric memory alone [28].

Although multi-agent systems may involve multiple roles, the simplest effective configuration consists of a *solver* and a *critic*. Accordingly, we focus our investigation on the critic, while treating the solver as a specialised LLM, a setting that has been extensively explored in prior work as discussed in the following.

#### 2.2.1 Critic: LLM as a judge

**LLM-as-a-Judge in Multi-Agent Systems.** Within multi-agent systems, the *LLM-as-a-Judge* paradigm has emerged as a powerful and versatile mechanism for coordinating, evaluating, and improving agent behaviour [24]. Its utility can be systematically understood along the following dimensions:

- **Evaluation of Generative Quality.** In text generation and open-ended response tasks, LLMs have been employed as judges to assess the overall quality of generated outputs via debate-based or comparative evaluation frameworks. For instance, Chan et al. [29] introduce a multi-agent debate setting in which autonomous agents argue over candidate responses, while a judge LLM determines the most compelling outcome. Similarly, Moniri et al. [30] propose an automated debate framework in which an LLM evaluator assesses domain knowledge, problem formulation, and logical consistency.
- **Reasoning and Decision Selection.** For reasoning-intensive tasks, judge LLMs play a central role in selecting or aggregating agent outputs. Liang et al. [31] present a multi-agent debating framework in which a judge LLM identifies the most reasonable answer among competing agents. Li et al. [32] further demonstrate that integrating a judge LLM within a layered multi-agent collaboration pipeline can improve both response quality and computational efficiency.
- **Assessment of Social and Interactive Dynamics.** Beyond task-level evaluation, LLMs have also been used to judge the quality of inter-agent social interactions. Zhang et al. [33] introduce the “Agent-as-a-Judge” framework, in which LLMs are instructed to act as evaluators of multi-agent social behaviour, enabling scalable assessment of cooperation, coordination, and interaction quality.
- **Domain-Specific Evaluation and Feedback.** In specialised domains, judge LLMs provide contextualised and nuanced feedback that would be difficult to encode using fixed metrics. In the financial domain, for example, Yu et al. [34] propose FinCon, a multi-agent system that leverages conceptual verbal reinforcement from an LLM-based evaluator to support and refine financial decision-making processes.

**Implications of LLM-as-a-Judge for Scalability and Optimisation.** More generally, the LLM-as-a-Judge paradigm is emerging as a key enabler of scalable AI development, especially as contemporary systems increasingly adopt interactive, iterative, and feedback-centric training methodologies [24]. In multi-agent environments, a judge LLM can continuously assess the effectiveness of inter-agent communication and coordination, providing detailed and adaptive feedback that surpasses the expressiveness of fixed evaluation metrics. When embedded within optimisation pipelines, these evaluators can steer model updates, strengthen reasoning processes, and facilitate adaptive coordination across agents [24]. Finally, LLM-based judges are not only evaluators of multi-agent behaviour but also tools for further optimising LLMs themselves. Recent work [35] demonstrates the integration of LLM-as-a-Judge mechanisms into multi-agent optimisation frameworks, where evaluative feedback guides agent interactions and learning dynamics. These results highlight the promise of judge LLMs as a scalable, general-purpose mechanism for enhancing decision quality, coordination efficiency, and overall system performance in multi-agent environments.

#### 2.2.2 Training strategy

With knowledge of single agent roles, it still remains a challenge to train the whole system overall. Training multi-agent LLM systems involves challenges beyond model optimisation, including agent initialisation, communication learning, credit assignment, cooperative training paradigms, and the integration of diverse supervision signals. There are several existing strategies, which we lay out in the following.

**Role-conditioned initialisation and parameter sharing.** The simplest approach, derived from single LLM training, is to initialise each agent from the same pretrained LLM and induce specialisation through role-conditioned prompts or adapters. For example, CAMEL [23] initialises each agent using role-playing prompts while sharing model parameters. AutoGen [36] adopts a similar strategy. AutoInit [37] further demonstrates that careful initialisation can improve multi-agent performance. This approach has the advantage of saving time compared to training a multi-agent system from scratch. However, merely initialising agents and sharing parameters without subsequent system-level fine-tuning limits the ability of agents to acquire task-specific knowledge and adapt collaboratively.

**System-level reward and credit assignment.** When it comes to system training, supervised multi-agent fine-tuning is applied to individual agents using their respective ground truth [38]. In contrast, reinforcement learning is more commonly employed because the contribution of a single agent to the final system performance is often not directly observable. In this context, the overall system performance naturally serves as the reward signal, making RL well suited for credit assignment. Techniques such as difference rewards can explicitly attribute the impact of each agent, providing a clear and effective mechanism for training multi-agent systems.

As discussed above, multi-agent systems offer a natural advantage in credit assignment. For instance, MAGRPO [39] employs centralised training with decentralised execution while assigning distinct objectives to different agents. Similarly, MAPoRL [40] implements systematic reward allocation that aligns with this principle. By providing appropriate credit assignment, these approaches enable agents to more effectively learn specialised capabilities within the multi-agent system.

**Cooperation or confrontation in self-play.** At a higher level than single-agent training objectives, multi-agent systems can employ systematic training strategies such as self-play, in which the system trains itself in an uninterrupted loop. During this process, agents may exhibit cooperative or competitive behaviours. For example, MARS [41] demonstrates multiple LLM agents interacting in games or reasoning tasks, while debate-based frameworks allow agents to critique one another to improve overall system output [42]. However, self-play approaches often suffer from a lack of external supervision within the loop, which can lead to drift or suboptimal revisions, as discussed previously.

#### 2.2.3 Human in the loop

Human-in-the-loop approaches are therefore well suited to address the lack of supervision in self-play or unsupervised multi-agent training. Active learning provides a framework for incorporating humans into the training loop, allowing timely feedback to guide model updates and improve performance [43].

There are several approaches to incorporating human-in-the-loop paradigms in LLM training. The explicit approach involves requesting human feedback during the training loop to revise LLM outputs. For instance, in machine translation, humans provide corrections or evaluations to guide model refinement [44], while in robotics, humans can provide heuristic instructions for path planning [45]. An implicit approach, which is widely adopted in reinforcement learning for LLMs, is the use of Proximal Policy Optimisation (PPO) [46], where human preferences serve as a reward signal to instruct the model’s learning process.

The integration of humans in the loop for multi-agent systems is an emerging research area, with relatively few studies focusing specifically on digital health applications. M3HF [47] provides a notable example, leveraging concepts analogous to those used by single large language models in reward shaping. Similarly, HARP [48] facilitates human-guided agent grouping, enabling non-experts to provide effective feedback with minimal intervention. There is also a theory that discusses the emergence of human-in-the-loop approaches in multi-agent systems [49], but it does not co-appear with experiments validating the effectiveness. However, in the context of digital health, critical challenges remain, particularly regarding dataset curation, model training, and ensuring that human feedback is reliably aligned with the system’s critic. Addressing these issues to enhance reliability and adaptability represents an important direction for future research.

### 2.3 Application in digital health

Multi-agent systems, as discussed above, can be adopted to solve a diverse range of tasks, amongst which lie a multitude of digital health applications. Although human involvement has not been emphasised in previous literature, which constitutes our main contribution to the proposed pipeline, existing applications already indicate that multi-agent systems have strong potential for future development in this domain.

#### 2.3.1 Role specialisation in clinical flow

Similar to other application domains, digital health involves complex processes that can be decomposed into multiple stages, where multi-agent systems naturally support role specialisation [50]. For example, MedCoAct is a multi-agent framework for clinical decision-making that incorporates distinct agents representing doctors and pharmacists, both of which play essential roles in reaching final decisions [51]. KG4Diagnosis adopts a similar multi-agent paradigm, but places greater emphasis on knowledge graph–based integration to support each stage of the diagnostic process [38]. Nevertheless, there remains a gap in existing work regarding explicit role specialisation that incorporates human insight within multi-agent clinical systems, as the opacity of such systems creates significant barriers to clinical adoption when interpretability techniques fail to capture complex agent interactions [52], potentially limiting the perceived trustworthiness of AI systems among patients who express discomfort with autonomous clinical decisions where human oversight is not clearly defined [53].

#### 2.3.2 Safety and reliable decision-making

Multi-agent systems also seek to mitigate these issues by incorporating verification mechanisms that supervise the decision-making process. For instance, the Tiered Agentic Oversight framework introduces automated inter- and intra-tier communication and role-based supervision to evaluate and validate model decisions [54]. However, such automated oversight cannot fully replace human involvement in real-world clinical systems, as the knowledge and reasoning processes of LLM-based agents remain partially uncontrollable and difficult to explain. While recent studies have explored the potential of deploying LLMs as agents in clinical and healthcare settings, they also highlight challenges related to reliability, interpretability, and safety [55].

#### 2.3.3 Personalised support and explainability

Similar to single LLM–based approaches in digital health, multi-agent systems can also be leveraged for personalised support, with the added benefit of improved explainability through explicit knowledge integration. For example, the MindCare multi-agent architecture first extracts patient profiles from clinical records, then retrieves condition-specific evidence from a knowledge base via RAG, and finally leverages an LLM to generate tailored recommendations, which is personalised for specific patients [56]. Additionally, prior work has emphasised the importance of explainability in digital health systems and outlined future research directions in this area [57]. Building on this perspective, recent studies have highlighted human–agent interaction within multi-agent frameworks as a key mechanism for enhancing personalisation and explainability, thereby underscoring the necessity of maintaining a human-in-the-loop in digital health applications [58]. Nevertheless, such systems do not explicitly integrate human knowledge into the system architecture, but instead rely on human input primarily in the form of prompts, which provides only limited and short-term influence.

There are many other digital health applications, which follow similar trends as the ones mentioned above, but to breakthrough current challenges of utilising multiagent system, we need a new pipeline to integrate human efforts in the training strategy.

## 3 Methodology

**Figure 2:**
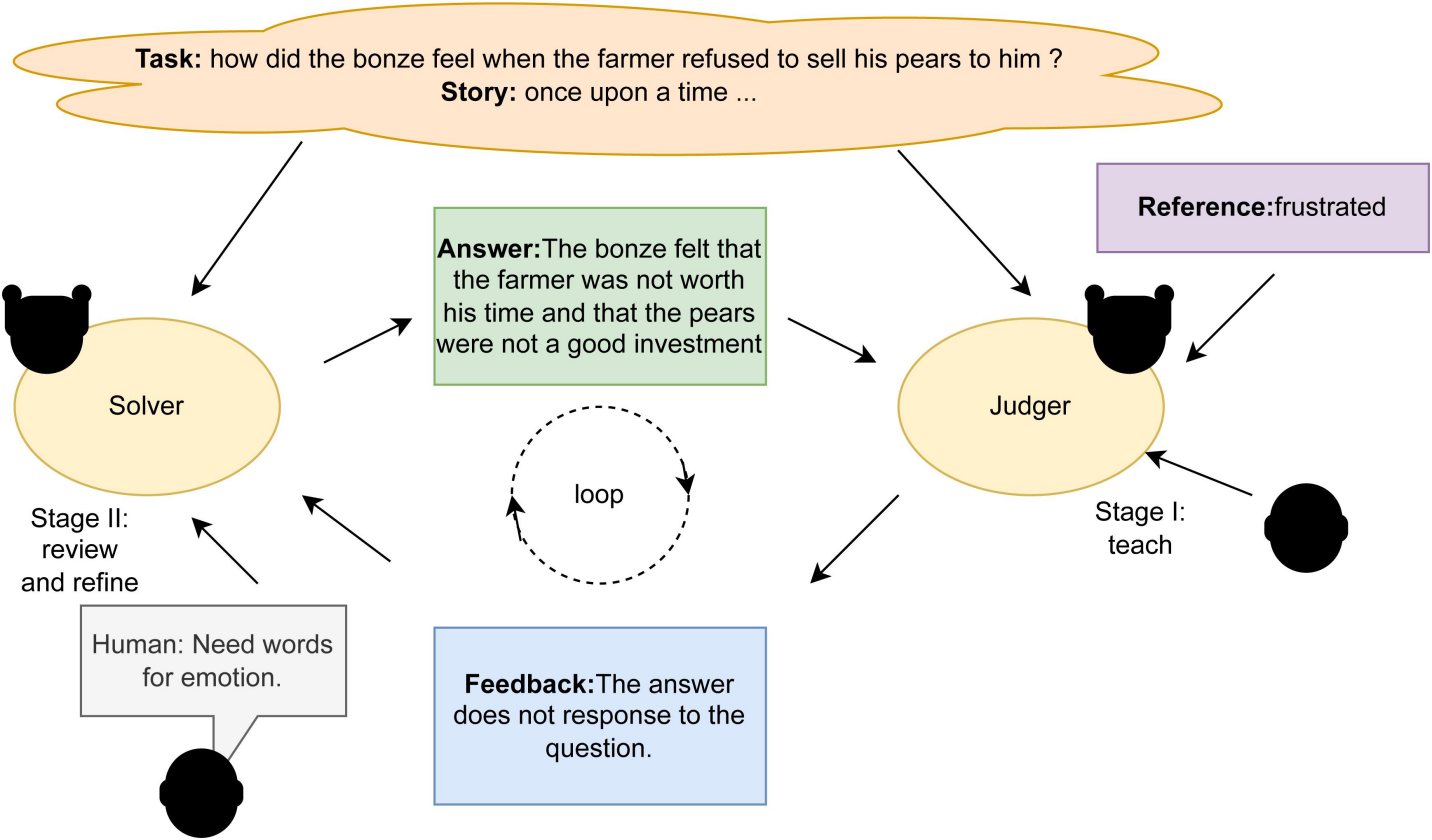
Overview of HAMAgent, a human-assisted multi-agent pipeline. It consists of two LLM agents: a solver that generates answers and a judge that provides feedback for improvement. The process iterates until a predefined stopping condition. Humans assist in two stages: (1) guiding the judge to produce better feedback (teaching stage), and (2) reviewing and refining the feedback to enhance the solver’s output quality.

We perform early stage exploration with a human in the loop-pipeline for multi-agent interaction with digital health perspective. We have drafted the model pipeline in Figure 2.

The proposed pipeline is applicable to a wide range of tasks, as long as they can be formulated as prompts and processed within the human-assisted multi-agent (HAMAgent) loop. The loop comprises two LLM agents: a solver, which addresses the task, and a judge, which provides feedback to refine the solver’s output. This architecture follows the general design of multi-agent systems described in prior work [59]. However, unlike previous studies where multiple agents collaborate to solve a single problem and may incur substantial inefficiency if an early decision leads in the wrong direction, our approach introduces two stages of human assistance. These stages aim to enhance the efficiency and performance of the iterative loop, and to integrate human values through natural language interactions.

The first stage is to teach the judge what is high-quality feedback. To that end, we extract segments of the run-time logs that record feedback generated by the judge given in response to answers produced by the solver. Human annotators then label those recorded answers, thereby encoding human judgment and values, especially important for health-related knowlegde that involves emotion or human motivation. We subsequently fine-tune the judge via supervised learning, treating the annotated dataset as groundtruth and offline human assistance. The fine-tuned judge is optimised to predict human labels from the recorded feedback, enabling it to emulate human evaluative behaviour at inference time as shown in Equation 1:

$$L_{\text{SFT}}=\ell (P_{\theta }(x,c,s),y),$$ (1)

where x is the input to the model, s and c are the solver’s answer and context, y is the human-provided feedback, respectively. $P_{\theta }$ is the model’s prediction.

The second stage focuses on reviewing the judge’s feedback and organising the resulting comments into prompts that can be directly referenced by the solver. By converting evaluative feedback into explicit textual guidance, the system bridges human judgment and automated reasoning. This stage constitutes the key human-in-the-loop component, allowing human values and subjective criteria to be systematically incorporated into the iterative problem-solving process.

## 4 Experiment and results

As part of our preliminary exploration of human-in-the-loop mechanisms within different components of a multi-agent framework particularly in the context of digital health, we conducted an initial trial to assess the effectiveness of the proposed architecture. For this purpose, we employed lightweight models, using DeepSeek-R1-distill-1.5B [60] as the solver and Qwen2.5-0.5B [61] as the judge. These models were selected because they are strong general-purpose reasoning LLMs without any specialised training in digital health. This choice allows us to evaluate whether the internal dynamics of the multi-agent system function as intended and to quantify the contribution of human expertise within the loop.

As a close representative of digital health, we focus on emotion recognition and related tasks that require genuine understanding of human values and are closely related to health-related topics, such as depression and general wellbeing [62]. However, there are currently no publicly available datasets involving actual patient-based data that can effectively be used. Specifically, we select the implicit question set portion of the FairytaleQA dataset [63], whose answer cannot be retrieved from the text directly such as checking the name of certain people, or the time certain events happen. This subset requires deep comprehension of the stories and includes questions such as identifying the emotions of characters. We do not directly use patient data due to the lack of publicly available datasets that require implicit emotional and contextual reasoning while meeting ethical and privacy constraints. However, this dataset is inferring characters’ emotions and internal states closely mimics affective reasoning demands in digital health. This design naturally extends to emotion-centered digital health applications (e.g., mental health) when suitable patient datasets become available. We use the provided training set for model training and the testing set for evaluation. Additionally, to prepare the data for the teaching stage in the HAMAgent framework, we randomly sample 100 examples from the validation set and manually annotate the solver’s answers, which is provided in the supplementary material. To prevent information leakage through the feedback, we ensure that the annotated feedback does not include any direct references to the correct answers. Instead, it only provides high-level hints that highlight where the solver’s output is problematic and how it could be improved.

The hyperparamters for training the judge are shown in Table 1.

**Table 1:** Training hyperparameters for the judge in the teaching stage.

Hyperparameter	Value
Batch size	4
Learning rate	2e−5
Epochs	20
Max length	1,024
Gradient accumulation steps	4

We report the judge’s evaluation using a rough matching score, which measures the overlap between the reference answers and the solver’s generated outputs. Specifically, ROUGE measures overlap between generated and reference text for evaluating summaries and translations. ROUGE-1 checks single-word matches, ROUGE-2 checks two-word phrase matches, and ROUGE-L finds the longest common subsequence to capture sentence structure. Higher scores indicate better alignment with reference texts. The prompt for the solver that we use is: *Story:s; Question:q; Previous Answer:a; Feedback:f; Now revise your answer based on the feedback. Improved Answer:*, and the prompt for judge is *Story:s; Question:q; Previous Answer:a; Groundtruth Answer:g; Please evaluate the answer and give detailed constructive feedback but do not show the correct answer in your feedback.* We use the performance after a single round of loop as the baseline, and we further evaluate a three-iteration loop as the multi-agent setting. Our HAMAgent results are reported alongside these baselines in Table 2. To perform a statistical results, we run the same experiments 5 times with different seeds.

**Table 2:** Rouge results (mean ± std over 5 runs).

Method	Rouge-1 ↑	Rouge-2 ↑	Rouge-L ↑
Single loop	.047±.005	.012±.003	.044±.005
Multi loop	.070±.006	.031±.007	.065±.004
Multi loop + teach	.068±.004	.015±.006	.060±.008
HAMAgent	.**132 ± .008**	.**041 ± .004**	.**073 ± .007**

HAMAgent significantly outperforms all baselines on all metrics (paired t-test, p<.001).

Bold values shows the best performing score.

Although this is an early and relatively simple exploration of the framework, the results reveal several promising findings. Despite the small model size and limited dataset, both contributing to the relatively low absolute performance scores, HAMAgent consistently achieves the best performance among all experimental settings. This highlights the importance of human intervention, especially in digital health applications where incorporating human values is essential. Moreover, the performance improvements observed from both the multi-loop teaching stage and the multi-loop solving stage further demonstrate the benefits of integrating humans into the loop at multiple points. The performance gains also validate the effectiveness of multi-agent self-evolution, where LLMs try to rethink the output of their previous answer and check its correctness.

We note that the multi-loop baseline slightly outperforms multi-loop + teach, which may appear counterintuitive given the additional supervision in the latter. However, this comparison should be interpreted with caution: the untuned judge in the multi-loop baseline is prone to reference leakage, inadvertently including ground truth content in its feedback to the solver, which artificially inflates its score. The teaching stage in multi-loop + teach specifically trains the judge to avoid such leakage, yielding a more honest but lower absolute score. Consequently, the two scores are not directly comparable on the same scale, and the apparent performance gap does not indicate a failure of the teaching mechanism. Rather, it highlights a known limitation of using untuned LLMs as evaluators in iterative feedback loops. Other factors such as context interference and prompt sensitivity may also contribute to the variance. This observation is consistent with previous studies on model jailbreaking [64]. For this reason, we did not experiment with a multi-loop setup that includes the second stage of human intervention. Moreover, to have a fair comparison of the baseline, we cannot apply more heuristic strategy other than strict filter of the prompt as this will be counted as human intervention. Importantly, HAMAgent still outperforms all baselines, demonstrating that our pipeline yields a more reliable system and improves overall performance.

In Table 3, we further provide one sample output for each framework.

**Table 3:** Case study of the full framework.

Method	Answer	Feedback	Final answer
Single loop	The other bannock felt that the bannock had been eaten and was not in good shape, and that the old woman had been watching for it and had seen it through the middle, leading to her deciding to eat it…	The given text describes an incident involving two bannocks eating a bannock. The other bannock was initially watching the first bannock for some time before deciding to eat it. … we can infer that the other bannock must have been scared.	–
Multi loop	The other bannock felt that the bannock had been eaten and was not in good shape, and that the old woman had been watching for it and had seen it through the middle, leading to her deciding to eat it…	The given text describes an incident involving two bannocks eating a bannock. The other bannock was initially watching the first bannock for some time before deciding to eat it. … we can infer that the other bannock must have been scared.	The other bannock felt very angry and upset.
Multi loop + teach	The other bannock felt that the bannock had been eaten and was not in good shape, and that the old woman had been watching for it and had seen it through the middle, leading to her deciding to eat it…	This is incorrect. It does not say what the bannock has been eating, just that it disappeared. Also there are more than one word in the sentence. The answer has 1 point out of 10.	The other bannock felt scared because the bannock had been eaten, which …
HAMAgent	The other bannock felt that the bannock had been eaten and was not in good shape, and that the old woman had been watching for it and had seen it through the middle, leading to her deciding to eat it…	It is the incorrect emotion. The story does not show positive attitude for previous content. Instead, it shows that it ran away. Try to think from human value perspective what the bannock feels when it decides to run away?	The other bannock felt it was too scared because the young woman was too eager to eat it.

It shows the solver’s initial answers, feedback from the judge, and the revised final answers. The story is *while they were toasting, her husband came in from the byre, and sat down to take a rest in his great arm-chair. presently his eyes fell on the bannocks, and, as they looked very good, he broke one through the middle and began to eat it. when the other bannock saw this it determined that it should not have the same fate, so it ran across the kitchen and out of the door as fast as it could. and when the old woman saw it disappearing, she ran after it as fast as her legs would carry her, holding her spindle in one hand and her distaff in the other.* with the question *how did the other bannock feel after it saw one of the banoocks get eaten ?* and reference answer *scared.*

It can be observed that the original judge may inadvertently leak the reference answers, whereas the taught judge is more likely to avoid disclosing the ground truth. Furthermore, the feedback provided by humans in HAMAgent is intuitively more heuristic. Additionally, large language models tend to generate more content than required, reflecting their inherent reasoning patterns, which may partially explain the relatively low ROUGE scores. Overall, these observations are consistent with the findings discussed earlier.

## 5 Implication and direction

Our preliminary experiments show that the HAMAgent framework has the potential to improve emotion recognition in digital health applications. LLMs can do reasoning, but their predictions of emotion recognition are often limited by training data, the lack of human values and the subjectiveness of the task itself. By incorporating human feedback, the system not only corrects errors but also benefits from human knowledge, experience, and contextual understanding that the model alone cannot capture. This combination may lead to more reliable detection of patients’ emotional states, which is critical for effective monitoring and intervention.

Moreover, human guidance allows for more perspective in the analysis of emotional data. LLMs inherently expose their reasoning process and may generate multiple possible outputs, which provides opportunities for diverse interpretations. Humans can select and refine these outputs to ensure they align with the patient’s specific situation.

Additionally, while this framework is demonstrated for emotion recognition, it can likely be generalised to other digital health tasks. Applications such as symptom triage, personalised recommendations, and mental health interventions can benefit from iterative human–AI feedback loops. By combining AI efficiency with human judgment, these loops can improve accuracy, ensure ethical compliance, and contribute to reliability of automated health support systems. This highlights the broader potential of human-in-the-loop AI in diverse healthcare contexts.

While our current work focuses on text-based emotion reasoning as a first step toward realizing HITL multi-agent systems in digital health, we recognize that real-world clinical and affective computing applications increasingly require multimodal integration. Recent efforts linking physiological and neurobiological biomarkers with LLMs have demonstrated complementary pathways beyond text-only reasoning. In the domain of EEG foundation models, LaBraM [65] learns generalizable EEG representations through large-scale pretraining, while Neuro-GPT [66] introduces a foundation model architecture specifically designed for EEG analysis. NeuroLM [67] further unifies diverse EEG tasks within a single LLM through cross-modal token alignment and instruction tuning, demonstrating the feasibility of bridging neural signals and language modeling. In neural decoding, MindGPT [68] translates perceived visual stimuli from fMRI signals into natural language descriptions, BrainLLM [69] directly incorporates brain representations into the language generation process, and BP-GPT [70] leverages fMRI-derived brain prompts to guide auditory neural decoding. More directly related to digital health, EEG Emotion Copilot [71] fine-tunes a lightweight LLM to interpret emotion-related EEG signals and generate supportive clinical records, demonstrating the potential for connecting physiological signals with clinical decision support.

Collectively, these studies highlight the value of multimodal frameworks in capturing affective and cognitive states that may not be fully revealed through textual information alone. The HITL feedback mechanism in HAMAgent can be naturally extended to such settings, where human experts are responsible not only for evaluating textual outputs but also for assessing cross-modal alignment quality and the clinical plausibility of multimodal reasoning processes. We therefore identify multimodal extension as an important direction for future research, with the potential to further enhance the robustness, interpretability, and clinical applicability of HITL multi-agent systems in digital health.

Besides the aforementioned aspects, an important future research direction is to scale up both the model and the dataset size. In this work, we relied on relatively small scale models and datasets for emotion recognition. Future studies could explore larger LMs and incorporate more diverse emotion-labelled datasets to improve robustness and generalisation. Moreover, this scaling can be extended beyond emotion recognition to other digital health applications, including multimodal settings that combine text, speech, and physiological signals. Such expansions could enable the framework to better handle complex, real-world healthcare data and support a wider range of patient monitoring and intervention tasks.

Moreover, we could design more efficient integration of human values into the framework. In the current setup, human intervention is applied at every stage, which can be time-consuming and resource-intensive, especially for the second stage. Future work could explore strategies where human feedback is incorporated selectively at key stages or during specific training phases, rather than continuously throughout the entire process. Such an approach would reduce human effort while still maintaining the benefits of human-guided corrections and value alignment, making the framework more practical for large-scale deployment in digital health applications. For example, transitioning from full human review to partial sampling (e.g., reviewing only 10% of cases) once the system reaches a stable self-iteration state—as well as distillation-based approaches that progressively replace human annotations with model-generated feedback.

Necessarily, we have to address privacy and safety concerns. Multi-turn interactions with LLMs can become more difficult to control than single LLMs, raising the risk of unintended information leakage. While minor leaks may be acceptable in general applications, digital health scenarios require stringent protection of sensitive patient data. Future research should focus on developing robust mechanisms to prevent privacy breaches, including secure model architectures, and controlled feedback loops, etc., ensuring that human-in-the-loop systems remain safe and compliant in healthcare settings.

Additionally, we could explore more diverse model architectures and evaluation strategies. The current framework primarily focuses on prediction tasks, but digital health often involves more complex outputs, such as report generation or detailed reasoning tasks. Due to the variability of natural language and the specific requirements of healthcare, future work could incorporate weighted scoring metrics that evaluate not only overall correctness but also clinically important aspects, such as diagnostic accuracy, reasoning validity, or explanation quality. Such enhancements would allow more comprehensive assessment and optimisation of human-in-the-loop systems in real-world healthcare applications.

## 6 Conclusion

This study explored the integration of human-in-the-loop feedback in multiagent systems for emotion and human behaviour understanding within digital health contexts. By leveraging the FairytaleQA dataset, we proposed the HAMAgent framework to assess how human feedback influences multi-agent reasoning and system performance. Our preliminary results suggest that incorporating human guidance into multi-LLM workflows enhances their effectiveness and adaptability, offering a promising avenue for digital health applications. Future work will focus on expanding our approach by incorporating additional datasets to test the framework’s robustness across diverse scenarios. Specifically, this work requires more datasets that are directly related to digital health, which is one of the limitations of the work. We also aim to explore a wider range of real-world digital health applications to fully assess the potential of human-in-the-loop systems in improving healthcare outcomes.

## Data Availability

The datasets presented in this study can be found in online repositories. The names of the repository/repositories and accession number(s) can be found in the article/Supplementary Material.
